# *Plasmodium berghei* ANKA causes intestinal malaria associated with dysbiosis

**DOI:** 10.1038/srep15699

**Published:** 2015-10-27

**Authors:** Tomoyo Taniguchi, Eiji Miyauchi, Shota Nakamura, Makoto Hirai, Kazutomo Suzue, Takashi Imai, Takahiro Nomura, Tadashi Handa, Hiroko Okada, Chikako Shimokawa, Risa Onishi, Alex Olia, Jun Hirata, Haruyoshi Tomita, Hiroshi Ohno, Toshihiro Horii, Hajime Hisaeda

**Affiliations:** 1 Department of Parasitology, Graduate School of Medicine, Gunma University, 3-39-22, Showa-machi, Maebashi, Gunma 371-8511, Japan; 2 Center for Medical Education, Graduate School of Medicine, Gunma University, 3-39-22, Showa-machi, Maebashi, Gunma 371-8511, Japan; 3 Laboratory for Intestinal Ecosystem, RIKEN Center for Integrative Medical Sciences (IMS), Yokohama, Kanagawa 230-0045, Japan; 4 Department of Genome Informatics, Research Institute for Microbial Diseases, Osaka University, Suita, Osaka 565-0871, Japan; 5 Department of Molecular and Cellular Parasitology, Juntendo University School of Medicine, Hongo, Bunkyo, Tokyo 113-8421, Japan; 6 Department of Bacteriology and Laboratory of Bacterial Drug Resistance, Gunma University Graduate School of Medicine, 3-39-22, Showa-machi, Maebashi, Gunma 371-8511, Japan; 7 Department of Diagnostic Pathology, Gunma University Graduate School of Medicine, 3-19-22, Showa-machi, Maebashi, Gunma, 371-8511, Japan; 8 Department of Molecular Protozoology, Research Institute for Microbial Diseases, Osaka University, Suita, Osaka 565-0871, Japan

## Abstract

Gastrointestinal symptoms, such as abdominal pain and diarrhea, are frequently observed in patients with *Plasmodium falciparum* malaria. However, the correlation between malaria intestinal pathology and intestinal microbiota has not been investigated. In the present study, infection of C57BL/6 mice with *P*. *berghei* ANKA (*Pb*A) caused intestinal pathological changes, such as detachment of epithelia in the small intestines and increased intestinal permeability, which correlated with development with experimental cerebral malaria (ECM). Notably, an apparent dysbiosis occurred, characterized by a reduction of Firmicutes and an increase in Proteobacteria. Furthermore, some genera of microbiota correlated with parasite growth and/or ECM development. By contrast, BALB/c mice are resistant to ECM and exhibit milder intestinal pathology and dysbiosis. These results indicate that the severity of cerebral and intestinal pathology coincides with the degree of alteration in microbiota. This is the first report demonstrating that malaria affects intestinal microbiota and causes dysbiosis.

Malaria caused by protozoan parasites of the genus *Plasmodium* is the most prevalent infectious disease in tropical and subtropical regions. Approximately half of the worldwide population is at risk of infection, and more than 200 million people are currently infected; the annual mortality rate is approximately 60 million people^1^. Malaria vaccines are urgently needed, but no effective malaria vaccine is yet available^2^. The pathogenesis of malaria is very complicated; it depends on parasite growth and host immunity to malaria parasites. Cerebral malaria is a lethal complication of falciparum malaria that depends on host immune responses^3^. Therefore, the elucidation of host protective mechanisms against malaria and parasite-host interactions is essential to strategies to control malaria, including vaccine development.

In addition to the malarial triad of fever, anemia, and splenomegaly, gastrointestinal symptoms, such as abdominal pain, diarrhea, and vomiting, develop in patients with falciparum malaria^3^,^4^. Pathological changes, observed mainly in the small intestine, include tongue-shaped villi of the jejunum and duodenum^5^, width extension and shortening of villi^6^, and bleeding due to ruptures of the villi or other mucosal components^7^. Malaria infections affect the intestinal tract, and changes in the intestinal environment appear to influence the pathogenesis of malaria. For example, superinfection of malaria patients with intestinal pathogens such as non-typhoidal *Salmonella*^8^ or helminthes^9^ have been shown to increase the severity of malaria symptoms. We also found that mice infected with the rodent malaria parasite *P. yoelii* 17XNL succumbed to otherwise non-lethal infection when mice were co-infected with an intestinal helminth *Heligmosomoides polygyrus*^10^. Therefore, it is likely that immune responses evoked in intestines may affect host defense mechanisms to malaria.

Recently, intestinal environments, especially alterations in intestinal microbiota, were shown to be associated with the onset of several diseases, including metabolic diseases^11^,^12^,^13^, such as obesity and diabetes, and immune disorders, such as autoimmune diseases^14^,^15^,^16^ and allergies^17^,^18^. Intestinal microbiota also modify the pathogenesis of infectious diseases, such as influenza^19^,^20^,^21^, which suggests that variability in intestinal microbiota influences systemic immune responses. Regulation of the immune system via modulation of intestinal microbes is partially explained by observations that certain bacteria activate specific types of immune cells. For example, segmented filamentous bacteria activate Th17 cells^22^ and some Clostridia species activate regulatory T cells (Treg)^23^,^24^,^25^. Furthermore, the gut–brain axis, which allows intestinal microbiota to stimulate the central nervous system, may be involved during the development of experimental autoimmune encephalomyelitis, a murine model of multiple sclerosis^14^, and psychiatric disorders, such as schizophrenia^26^ and autism^27^.

Given the evidence described above, we speculated that severe malaria affecting the central nervous system and intestines might alter the intestinal microbiota. However, it remains unknown whether intestinal microbiota are involved in host-parasite interactions during malaria. In this study, we investigated the intestinal pathology and changes in intestinal microbiota following infection with *P. berghei* ANKA (*Pb*A), which causes experimental cerebral malaria (ECM) in C57BL/6 (B6) mice but not insensitive BALB/c mice. Our results revealed that intestinal pathology was more severe in B6 mice infected with *Pb*A and was associated with marked changes in microbiota compared with BALB/c mice. These results support the involvement of intestinal microbiota in host-parasite interactions during malaria infection.

## Results

### Pathological changes in the intestinal tract in *Pb*A-infected mice

**Figure 1:**
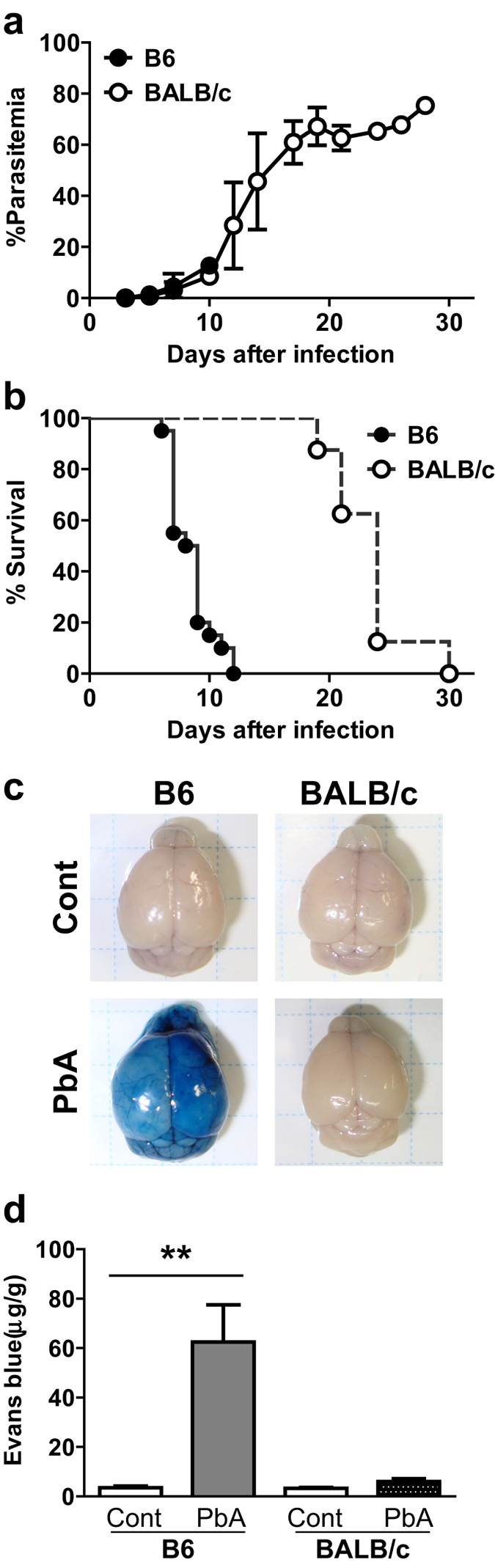
Differences in pathology between B6 and BALB/c mice infected with *Pb*A. Parasitemia (**a**) and survival (**b**) were monitored in B6 and BALB/c mice infected with *Pb*A. Parasitemia values are means ± S.D. from 20 B6 and 15 BALB/c mice. (**c,d**) Mice were injected with Evans blue dye 9 days after infection. Uninfected mice were used as controls. Brains of mice (**c**) and amounts of EB per g of brain (**d**) are shown. Data are means ± S.D. from 5 mice. Three repeated experiments showed similar results. Statistical analysis was performed using the Mann–Whitney *U*-test. **p < 0.05.

The rodent malaria parasite *Pb*A provides a good model for ECM. B6 mice infected with *Pb*A developed neurological symptoms, such as ataxia, convulsions, and paralysis, and died within 2 weeks, despite low parasitemia (Fig. 1a,b). These mice exhibited a collapse of the blood–brain barrier, as revealed by leakage of intravenously injected Evans blue (EB) dye into the brain parenchyma (Fig. 1c,d), indicating that the development of ECM led directly to the observed lethality. By contrast, *Pb*A-infected BALB/c mice began to die 3 weeks after infection, but their death was associated with high parasitemia (Fig. 1a,b). Infection of BALB/c mice with *Pb*A did not cause EB leakage, which suggests the absence of ECM (Fig. 1c,d) and confirms that the pathologies in the B6 and BALB/c mice were different.

**Figure 2:**
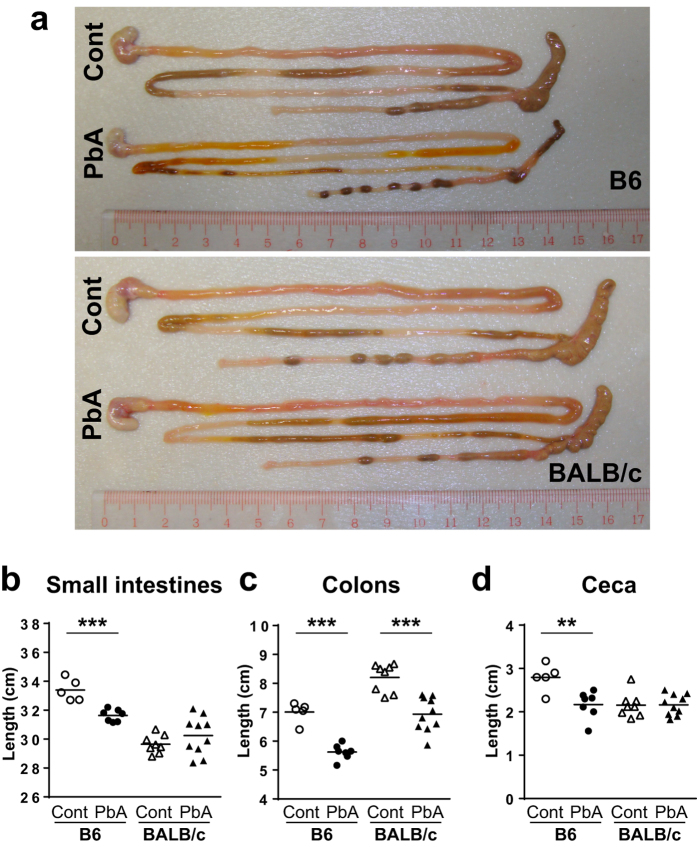
Appearance of intestines in mice infected with *Pb*A. Representative photographs of whole intestines (**a**) and the length of small intestines (**b**) colons (**c**) and cecum (**d**) from *Pb*A-infected B6 and BALB/c mice at 9 days after infection are shown. Uninfected mice were used as controls. Each symbol represents an individual mouse, and bars indicate the mean of 5–10 mice. Three repeated experiments showed similar results. Statistical analysis was performed using Student’s unpaired *t*-test. **p < 0.01, ***p < 0.001.

Next, we evaluated the effect of *Pb*A infection on the intestinal tract at 9 days after infection, when ECM had developed in B6 mice. Macroscopically, infected B6 mice appeared to have less intestinal content in the cecum and lower areas of the intestine than uninfected control mice did (Fig. 2a). Whole intestines, including the small intestines, cecum, and colon, were significantly shortened after *Pb*A infection in B6 mice (Fig. 2a–d). No significant changes in intestinal appearance were observed in BALB/c mice, except for a shortening of the colon (Fig. 2a–d).

**Figure 3:**
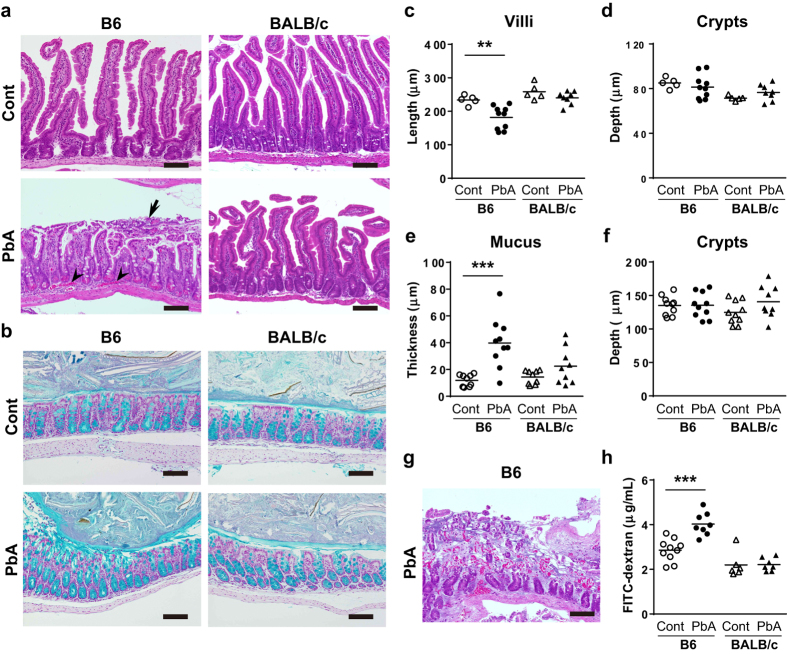
Histological analyses of the small intestine in mice infected with *Pb*A. Small or large intestines from B6 and BALB/c mice that were uninfected or 9 days after infection were stained with H&E (**a,g**) or alcian blue and nuclear fast red (**b**) respectively. Detachment of the intestinal epithelia (arrow) and sequestration of red blood cells in blood vessels (arrowheads) were observed. Length of villus (**c**) and depth of crypt (**d**) in the small intestine were measured. Each symbol indicates the mean of 100 villi or crypts from an individual mouse. Bars indicate the means of more than 5 mice. Thickness of mucus (**e**) and depth of crypts (f) in the colon were measured. Each symbol indicates the mean of 30 to 80 areas of mucus or 15 to 50 crypts from an individual mouse. (**g**) Representative micrographs of H&E-stained intestinal sections from *Pb*A-infected B6 mice indicate the destruction of villi and microscopic hemorrhage. (**h**) FITC-dextran concentrations in serum were measured in B6 mice that were uninfected or at 9 days after infection with *Pb*A. Each symbol indicates an individual mouse. Bars indicate the means of 5 or 8 control or *Pb*A-infected mice, respectively, from one experiment representative of the three experiments performed. Scale bars = 100 μm. Statistical analysis was performed using Student’s unpaired *t*-test. **p < 0.01, ***p < 0.001.

We next conducted a histological assessment of the intestines because shortening of the intestines is indicative of inflammation. In infected B6 mice, villi in the small intestines were remarkably shortened, but the depth of crypts was not altered (Fig. 3a,c,d). Detachment of intestinal epithelia and occlusion of blood vessels were also observed, presumably due to the adhesion of red blood cells in intestinal submucosal areas (Fig. 3a). In most of the mice, destruction of villi and microscopic bleeding were also observed (Fig. 3g). Intestinal permeability was increased, as determined by the detection of higher serum concentrations of orally administered FITC-dextran due to enhanced transition from the intestinal lumen to the blood (Fig. 3h). An increase in intestinal permeability is consistent with human cases of severe falciparum malaria^28^. Together with the shortening of intestines, these results indicate that pathological events occurred in the small intestines in B6 mice infected with *Pb*A. Although a shortening of the colons was observed, there were no inflammatory changes there. Only a thickened mucus layer stained with alcian blue was observed in the colons (Fig. 3b,e,f). As in the small intestines, the depth of crypts was not affected (Fig. 3f). No histological changes in whole intestines were observed in BALB/c mice (Fig. 3a–f,h). However, a shortening of the colons was observed (Fig. 2a,c).

### Dysbiosis in *Pb*A-infected mice

**Figure 4:**
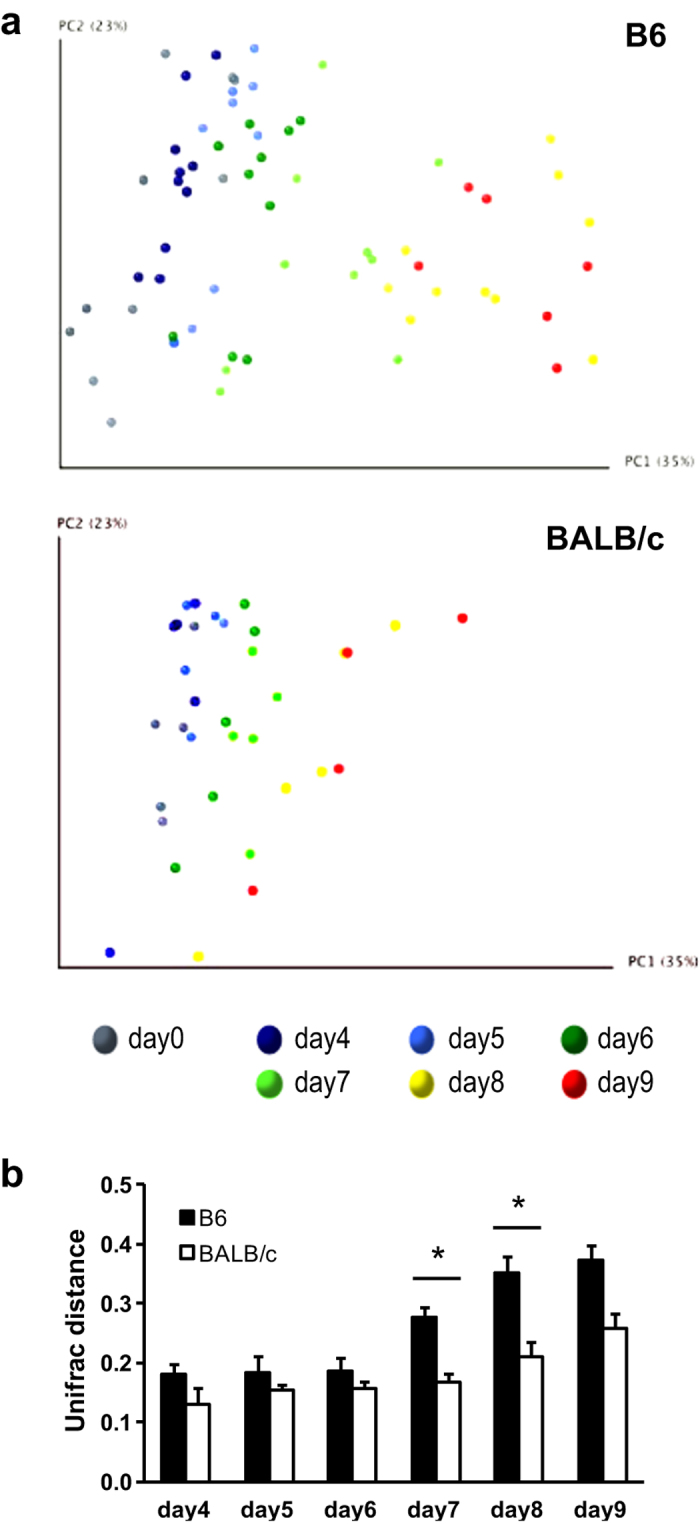
Temporal changes in intestinal microbiota in mice infected with *Pb*A. (**a**) Principal coordinate analyses based on weighted UniFrac distance of fecal microbiota from B6 (n = 10) and BALB/c (n = 5) mice. Each symbol represents an individual mouse at different time periods and discriminated by different colors as indicated. (**b**) Mean weighted UniFrac distances between before and after *Pb*A infection in B6 and BALB/c mice are shown. Values are means ± S.D. from 10 B6 and 5 BALB/c mice. Statistical analysis was performed using two-way ANOVA with Bonferroni’s post hoc test. **p < 0.01 compared with uninfected mice.

Recent studies reported that the intestinal microbiota was altered in various diseases^11^,^12^,^13^,^14^,^15^,^16^,^17^,^18^,^19^,^20^,^21^,^22^,^23^,^26^,^27^, which led us to hypothesize that malaria affects the intestinal microbiota. To investigate this, we analyzed the composition of the intestinal microbiota by sequencing 16S rRNA gene amplicons obtained from the feces of infected mice using a next-generation sequencer. We identified 4,906,013 bacterial 16S rRNA gene sequences from 100 samples (9,619–348,401 reads per sample). The sequences were assigned to 4,714 species-level operational taxonomic units (OTUs). Alpha diversity metrics, including the number of observed OTUs, the Shannon and Simpson diversity index, and phylogenetic diversity revealed a trend that infection with *Pb*A transiently (day 5 to 7) increased richness and evenness, then decreased within 9 days (Supplementary Fig. 1). Principal coordinate analyses revealed that intestinal microbiota changed markedly as *Pb*A infection progressed in both B6 and BALB/c mice (Fig. 4a). Interestingly, significant changes in the microbiota occurred earlier and to a greater degree in B6 mice than in BALB/c mice (Fig. 4b).

**Figure 5:**
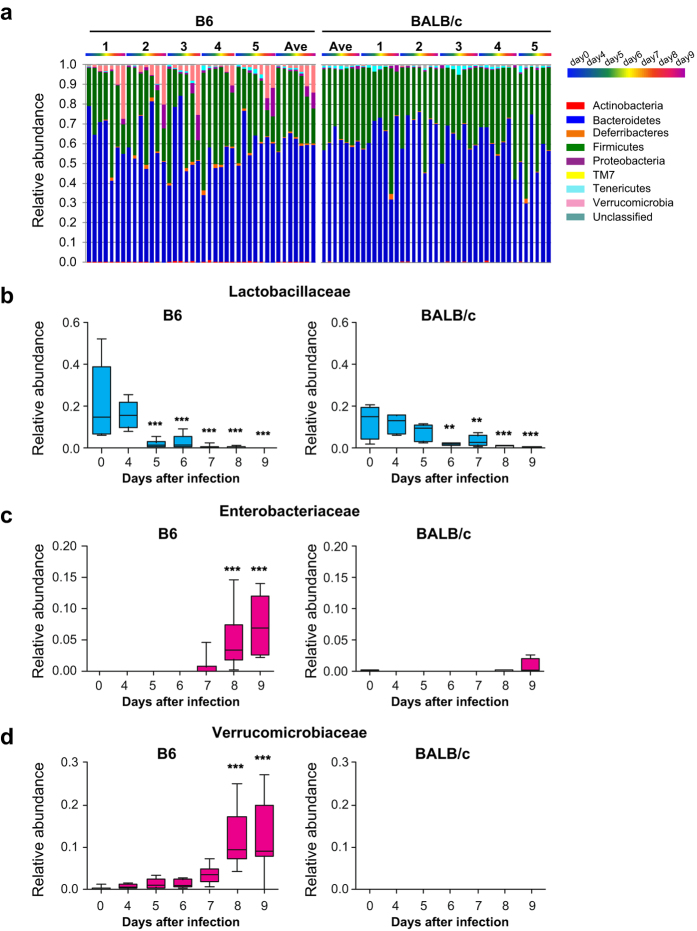
Temporal changes in intestinal bacterial components in mice infected with *Pb*A. (**a**) Relative abundance of fecal bacterial phyla in 5 B6 and 5 BALB/c mice. The numbers indicate individual mice, and each bar represents the results at the indicated time periods. (**b**–**d**) The relative abundance of the family of Lactobacillaceae (**b**), Enterobacteriaceae (**c**), and Verrucomicrobiaceae (**d**) in B6 and BALB/c mice infected with *Pb*A. Values are the means ± S.D. from 10 B6 and 5 BALB/c mice. Statistical analysis was performed using Dunnett’s test. **p < 0.01, ***p < 0.001 compared with uninfected mice.

Comparisons at the phylum level indicated that Bacteroidetes and Firmicutes were the major components of the intestinal microbiota before infection, regardless of the mouse strain (Fig. 5a). *Pb*A infection of B6 mice markedly reduced Firmicutes and increased Proteobacteria and Verrucomicrobia (Fig. 5a). A slight modulation was observed in BALB/c mice infected with *Pb*A (Fig. 4), but no alterations were observed at the phylum level. We further identified the families that were altered following infection. The Lactobacillaceae family, belonging to Firmicutes, was drastically reduced in B6 mice from day 5 and prior to the onset of neurological symptoms (Fig. 5b). Enterobacteriaceae in the Proteobacteria phylum and Verrucomicrobiaceae in the Verrucomicrobia phylum were also increased in B6 mice exhibiting neurological symptoms at 8 and 9 days after infection (Fig. 5c,d). A decrease in Lactobacillaceae was also observed in BALB/c mice that were refractory to ECM at 6 days after infection (Fig. 5b). However, increases in Enterobacteriaceae and Verrucomicrobiaceae were not observed.

### The correlation between *Pb*A pathogenicity and changes in microbiota

**Figure 6:**
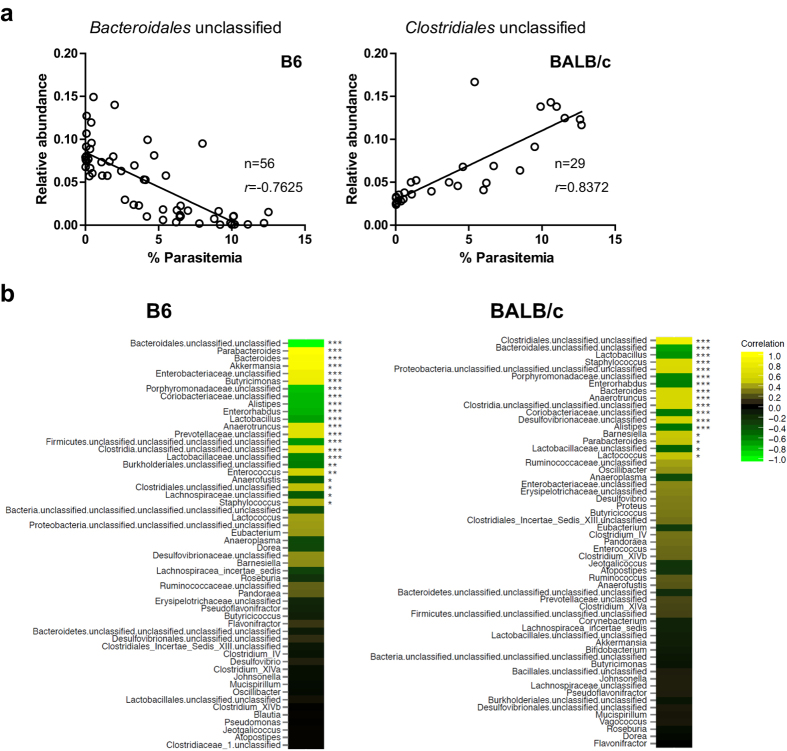
Correlation between genera of bacteria and parasite burden in mice infected with *Pb*A. (**a**) Representative co-plotted parasitemia and abundance of genera in 10 B6 and 5 BALB/c mice at 4 to 9 days after infection are shown. n and r denote the number of plots and the correlation coefficient, respectively. (**b**) The genera are listed in order of decreasing strength of correlation. Bright yellow or green indicates a stronger positive or negative correlation, respectively. Statistical analyses were performed using Pearson’s correlation coefficient test. *p < 0.05, **p < 0.01, ***p < 0.001.

To evaluate whether marked changes in the microbiota following *Pb*A infection were associated with malaria pathogenesis, we analyzed the abundance of microbes at the genus level and the percent parasitemia or cerebral malaria (CM) score, which indicates the severity of central nervous symptoms (Fig. 6a). In B6 mice, 22 genera were significantly correlated with parasitemia, with 11 genera showing a positive correlation and the other 11 showing a negative correlation (Fig. 6b). In BALB/c mice, 10 genera were positively correlated and 7 were negatively correlated with parasitemia (Fig. 6b). Thirteen genera were commonly found to be correlated in B6 and BALB/c mice. Furthermore, CM scores in B6 mice were correlated with 16 genera, 15 of which were also correlated with parasitemia (Supplementary Fig. 2). The genus *Lactobacillus* of the Lactobacillaceae family was decreased in both B6 and BALB/c mice and exhibited a strong inverse correlation with parasitemia and CM scores (Fig. 6). Verrucomicrobiaceae and Enterobacteriaceae family members were specifically increased in B6 mice (Fig. 5c,d), and *Akkerimansia* species and unclassified Enterobacteriaceae showed a sharp positive correlation with parasitemia and CM scores.

## Discussion

In this study we revealed that intestinal pathology occurred in a murine model of malaria. Pathological changes, including a shortening of the villi and bleeding in the small intestine, in *Pb*A-infected B6 mice were consistent with the histology of the small intestines in patients with *P. falciparum* malaria^6^,^7^,^29^,^30^. Gastrointestinal symptoms during falciparum malaria are thought to be caused by tumor necrosis factor (TNF)^31^, free radicals^32^, intestinal ischemia^32^, and digestive malabsorption of nutrients due to failure of the liver and pancreas^5^,^6^,^33^,^34^. However, the precise mechanisms remain unclear. High numbers of intestinal lesions caused by *Pb*A were observed in B6 mice susceptible to ECM, but not in resistant BALB/c mice, which suggests that intestinal pathology is associated with the pathogenicity of ECM. Indeed, the sequestration of red blood cells to blood vessels was observed only in the intestines of B6 mice (Fig. 3a).

16S rRNA gene sequencing of the microbiota clearly revealed that intestinal dysbiosis occurred in *Pb*A-infected mice. The degree of dysbiosis was much higher in B6 mice that exhibited cerebral and intestinal pathologies than in BALB/c mice that exhibited minimum pathological changes in brain and intestines. Therefore, dysbiosis and pathological changes are closely correlated. However, we did not address whether dysbiosis caused the pathological changes or whether it was secondary to malaria pathologies. For example, B6 mice about to develop ECM were very sick and ate very little. The lack of food in the intestines could affect the microbiota. *Pb*A-infected B6 mice contained less intestinal content than before infection (Fig. 2a). Inflammatory pathologies in the intestines caused by *Pb*A infection may alter the microbiota. Previous studies demonstrated the effects of inflammation on the intestinal microbiota in inflammatory bowel diseases^35^,^36^,^37^ and intestinal infections^38^,^39^. However, changes in the microbiota occurred prior to the development of intestinal pathologies in B6 mice, as early as 5 days after infection, and even in BALB/c mice that were free from intestinal pathologies. These results suggest that infection with *Pb*A itself causes dysbiosis.

Alternatively, dysbiosis may cause malaria pathologies. Two types of changes in microbiota occurred in our study. Some changes occurred in both B6 and BALB/c mice, whereas others occurred in only one mouse strain. For example, a reduction in Lactobacilliceae and an increase in Proteobacteria were observed in both mice strains, suggesting their involvement in the development of the common symptoms of malaria, such as elevated parasitemia. However, an increase in Enterobacteriaceae, known to be related to the development of colitis^40^, was obvious only in B6 mice, which may contribute to the observed intestinal pathologies via alterations in immune responses. *Akkermansia muciniphila* belonging to *Akkermansia* increased only in B6 mice. This bacteria degrades mucin, which is the main component of the mucus layer that functions as an intestinal barrier^41^,^42^. *Akkermansia* may be involved in the increased permeability of small intestines observed in B6 mice (Fig. 3e). However, *Akkermansia* normally resides in the colon and maintains the integrity of the mucus layers^41^,^42^. By contrast, Clostridia is known to activate intestinal Treg^23^, and some genera of unclassified Clostridiales increased only in BALB/c mice. These bacteria may be involved in the resistance to ECM by activating Treg^43^. However, these speculations must be experimentally proven using germ-free mice or stool-transfer experiments. Germ-free mice infected with the *P. berghei* NYU-2 strain, which did not cause ECM, showed development of similar level of parasitemia^44^. These results suggest a marginal effect of the microbiota on parasite development. The absence of microbiota also enhances antibody responses against malaria, which indicates that the microbiota affect immune responses to malaria parasites. It would be useful to determine the microbiotic influences on brain and intestinal pathologies and/or the immune responses that are responsible for pathogenicity in germ-free mice during *Pb*A infection.

Although we observed dysbiosis during malaria, it is not clear how malaria parasites interact with intestinal microbiota. The *in vivo* imaging of malaria parasites expressing luciferase demonstrated that they were abundant in the mesentery^45^. The presence of parasitized RBCs in the intestinal lumen must have been due to hemorrhage of the intestinal vessels (Fig. 3d). Therefore, these microbes may directly interact with one another. Indeed, intestinal microbiota affect the growth of parasites in *Anopheles* mosquitoes, a vector of malaria^46^. In addition, given that 70% of immune cells are distributed in gut-associated tissues, immune responses to malaria parasites may also affect the intestinal microbiota. Interactions between protozoa, microbiota, and host immunity may occur. Recently, it was reported that antibody responses to intestinal *Escherichia coli* O86:B7 suppressed malaria transmission^47^. Therefore, there is increasing evidence that intestinal environments, including the immune system, may affect host-parasite relationships during malaria.

In summary, we attempted to elucidate the influence of intestinal environments on host-pathogen interactions during malaria by focusing on intestinal pathology. We demonstrated, for the first time, marked changes in the intestinal microbiota following malaria infection. Further investigations of the relationship between dysbiosis and intestinal pathogenesis using our model will add to our understanding of the mechanisms involved in intestinal pathology in malaria patients.

## Methods

### Mice and parasites

Male B6 and BALB/c mice 6 to 12 weeks old were purchased from SLC (Hamamatsu, Japan) and maintained under specific pathogen-free conditions. Age- and sex-matched groups were used for the experiments. All animal experiments were reviewed and approved by the Committee for Ethics on Animal Experiments in the Graduate School of Gunma University (approval number 12–031), and were carried out in accordance with the approved guidelines for Animal Experiments in the Graduate School of Medicine, Gunma University, and the law (no. 105) and notification (no. 6) of the Japanese government.

*Pb*A was a generous gift from Dr. M. Torii (Ehime University, Japan). Blood-stage parasites for experimental infections were obtained from donor mice 5 days after inoculation with frozen stock. Mice were infected with 2.5 × 10^4^ parasitized red blood cells via intraperitoneal injection. Following infection, survival and parasitemia were monitored throughout the observation period. Parasitemia was assessed by determining the percentage of parasitized red blood cells in Giemsa-stained thin smears using blood from the tail vein under a microscope.

### Assessment of ECM and blood-brain barrier function

ECM was evaluated via observation of clinical signs, and disease severity was scored based on abnormal behavior as follows: 0, normal; 1, decrease in spontaneous activity; 2, loss of escape from handling; and 3, non-abdominal body position^48^,^49^. Blood–brain barrier function was assessed using EB dye as described previously^50^. Briefly, mice were injected intravenously with 2% EB (0.2 ml, Sigma Aldrich, St. Louis, MO, USA) and sacrificed and perfused with heparinized PBS 1 h later. Brains were surgically removed, weighed, and placed in 100% formamide (2 ml, Wako Pure Chemical Industries, Ltd, Osaka, Japan) for 48 h at 37 °C to extract the Evans blue dye. Absorbance was measured at 630 nm using an iMark microplate reader (Bio-Rad, Hercules, CA, USA). EB concentrations were measured from a standard curve, and the results were adjusted to the weight of the dye per g of brain tissue.

### Assessment of pathological changes in the intestines

Whole intestines were excised from *Pb*A-infected mice after perfusion at 9 days post-infection and photographed. The lengths of the small intestines, cecum, and colons were measured. Intestines were fixed in 10% phosphate-buffered formalin and embedded in paraffin. Sections (3 μm) of the small intestines were stained with hematoxylin and eosin (H&E). Dewaxed-3-μm-thick colon sections were washed with 3% acetic acid before and after staining with alcian blue, followed by counterstaining with nuclear fast red to evaluate the inner mucus layers. The specimens were observed using a BIOREVO BZ-9000 microscope (Keyence, Osaka, Japan).

Intestinal permeability was evaluated using a fluorescein isothiocyanate (FITC)-labeled dextran method, as previously described^27^. Mice were administered 4 kDa FITC-dextran (Sigma Aldrich) via oral gavage (0.6 mg/g body weight). Four hours later, serum samples were read for fluorescence measurements (488 nm excitation/530 nm emission) in triplicate using an EnSpire multimode plate reader (PerkinElmer, Inc., Waltham, MA, USA). Concentrations of FITC-dextran in sera were calculated from a standard curve.

### DNA extraction from stool samples

Stool samples collected from mice were immediately frozen using liquid nitrogen and stored at −80 °C until use. Bacterial genomic DNA was isolated as described previously with modifications^51^,^52^. The bacterial pellet was suspended and incubated with lysozyme (15 mg/ml, Wako Pure Chemical Industries, Ltd) at 37 °C for 1 h in 100 mM Tris-HCl/10 mM EDTA (10 × TE). Purified achromopeptidase (Wako Pure Chemical Industries, Ltd) was added at a final concentration of 2000 U/ml and then incubated at 37 °C for 30 min. The suspension was treated with 1% (wt/vol) sodium dodecyl sulfate (Wako Pure Chemical Industries, Ltd) and proteinase K (1 mg/ml, Merck Japan, Tokyo, Japan) and incubated at 55 °C for 1 h. The lysate was treated with phenol/chloroform/isoamyl alcohol, and DNA in the aqueous phase was precipitated by the addition of ethanol and pelleted via centrifugation at 5,000 × g at 4 °C for 15 min. The DNA pellet was rinsed with 75% ethanol, dried, and dissolved in 1 × TE. DNA samples were purified by treatment with RNase A (1 mg/ml, Wako Pure Chemical Industries, Ltd) at 37 °C for 30 min and precipitated by the addition of equal volumes of a 10% polyethylene glycol solution (PEG6000-2.5 M NaCl). DNA was pelleted via centrifugation at 18,000 × g at 4 °C for 10 min, rinsed with 75% ethanol, and dissolved in 1 × TE.

### Barcoded pyrosequencing analyses

The V4 variable region of the 16S ribosomal RNA (rRNA) gene was amplified by PCR using dual barcoded primers as described previously^53^. PCR amplicons were purified using AMPure XP magnetic purification beads (Beckman Coulter, Inc., Brea, CA, USA) and quantified using the Quant-iT PicoGreen ds DNA Assay Kit (Life Technologies Japan, Ltd, Tokyo, Japan). The pooled amplicons were sequenced using MiSeq (Illumine, Inc., San Diego, CA, USA) according to the manufacturer’s instructions.

### Data analysis

The 16S rRNA reads were processed using Mothur following the Mothur MiSeq SOP (http://www.mothur.org/wiki/MiSeq_SOP). In brief, the assembled reads were screened to eliminate reads that contained ambiguous bases and aligned to the SILVA 16S rRNA sequence database. Chimeric sequences were removed using Uchime^54^. The remaining reads were clustered into 97% identity Operational Taxonomic Units (OTUs) and assigned a taxonomy using the Ribosomal Database Project database (trainset9_032012.pds). The resulting OTU table was rarefied to 9,619 reads (the lowest read number among the samples), and estimations of alpha diversity metrics (phylogenetic diversity, observed OTU number, Chao1, Shannon’s index, and Simpson’s index) and weighted UniFrac analysis were performed in QIIME^55^. Correlation coefficients between bacterial abundance and parasitemia were calculated and visualized using the WGCNA R-package^56^. The pyrosequencing data are available at DDBJ under accession number BioProject: PRJDB3900 (PSUB004629) (http://trace.ddbj.nig.ac.jp/dra/index_e.html).

### Statistical analysis

Unpaired-*t* tests, Dunnett’s test, Mann–Whitney *U* test, one-way or two-way ANOVA and Bonferroni’s post-test were used, and all analyses were performed using Prism (GraphPad Software version 6.0, La Jolla, CA, USA).

## Additional Information

**How to cite this article**: Taniguchi, T. *et al*. *Plasmodium berghei* ANKA causes intestinal malaria associated with dysbiosis. *Sci. Rep*. **5**, 15699; doi: 10.1038/srep15699 (2015).

## Supplementary Material

Supplementary Information
