# Corrigendum: *Plasmodium berghei* ANKA causes intestinal malaria associated with dysbiosis

**DOI:** 10.1038/srep17248

**Published:** 2016-01-07

**Authors:** Tomoyo Taniguchi, Eiji Miyauchi, Shota Nakamura, Makoto Hirai, Kazutomo Suzue, Takashi Imai, Takahiro Nomura, Tadashi Handa, Hiroko Okada, Chikako Shimokawa, Risa Onishi, Alex Olia, Jun Hirata, Haruyoshi Tomita, Hiroshi Ohno, Toshihiro Horii, Hajime Hisaeda

Scientific Reports 5: Article number: 1569910.1038/srep15699; published online: 10272015; updated: 01072016.

This Article contains a typographical error in the Introduction.

“the annual mortality rate is approximately 60 million people^1^”

should read:

“the annual mortality rate is approximately 0.6 million people^1^”

