# Supplementary material for: Plasmodium berghei ANKA causes intestinal malaria associated with dysbiosis
Source: Sci Rep. 2015 Oct 27;5:15699. doi: 10.1038/srep15699 (PMC4621605; doi:10.1038/srep15699)
Supplement: Supplementary Information [file srep15699-s1.pdf]

Supplementary information

Plasmodium berghei ANKA causes intestinal malaria associated with dysbiosis

Tomoyo Taniguchi[1,2], Eiji Miyauchi[3], Shota Nakamura[4], Makoto Hirai[5], Kazutomo Suzue[1], Takashi Imai[1], Takahiro Nomura[6], Tadashi Handa[7], Hiroko Okada[1], Chikako Shimokawa[1], Risa Onishi[1], Alex Olia[1], Jun Hirata[1], Haruyoshi Tomita[6], Hiroshi Ohno[3], Toshihiro Horii[8] and Hajime Hisaeda[1*]

[1]Department of Parasitology, Graduate School of Medicine, Gunma University, 3-39-22, Showa-machi, Maebashi, Gunma 371-8511, Japan

[2]Center for Medical Education, Graduate School of Medicine, Gunma University, 3-39-22, Showa-machi, Maebashi, Gunma 371-8511, Japan

[3]Laboratory for Intestinal Ecosystem, RIKEN Center for Integrative Medical Sciences (IMS), Yokohama, Kanagawa 230-0045, Japan,

[4]Department of Genome Informatics, Research Institute for Microbial Diseases, Osaka University, Suita, Osaka 565-0871, Japan

[5]Department of Molecular and Cellular Parasitology, Juntendo University School of Medicine, Hongo, Bunkyo, Tokyo 113-8421, Japan

[6]Department of Bacteriology and Laboratory of Bacterial Drug Resistance, Gunma University Graduate School of Medicine, 3-39-22, Showa-machi, Maebashi, Gunma 371-8511, Japan

[7]Department of Diagnostic Pathology, Gunma University Graduate School of Medicine, 3-19-22, Showa-machi, Maebashi, Gunma, 371-8511, Japan

[8]Department of Molecular Protozoology, Research Institute for Microbial Diseases, Osaka University, Suita, Osaka 565-0871, Japan

*Corresponding author.
Department of Parasitology, Graduate School of Medicine, Gunma University Graduate School of Medicine, 3-39-22, Showa, Maebashi, Gunma 371-8511, Japan
E-mail: hisa@gunma-u.ac.jp (H. Hisaeda); Tel: +81-27-220-8023

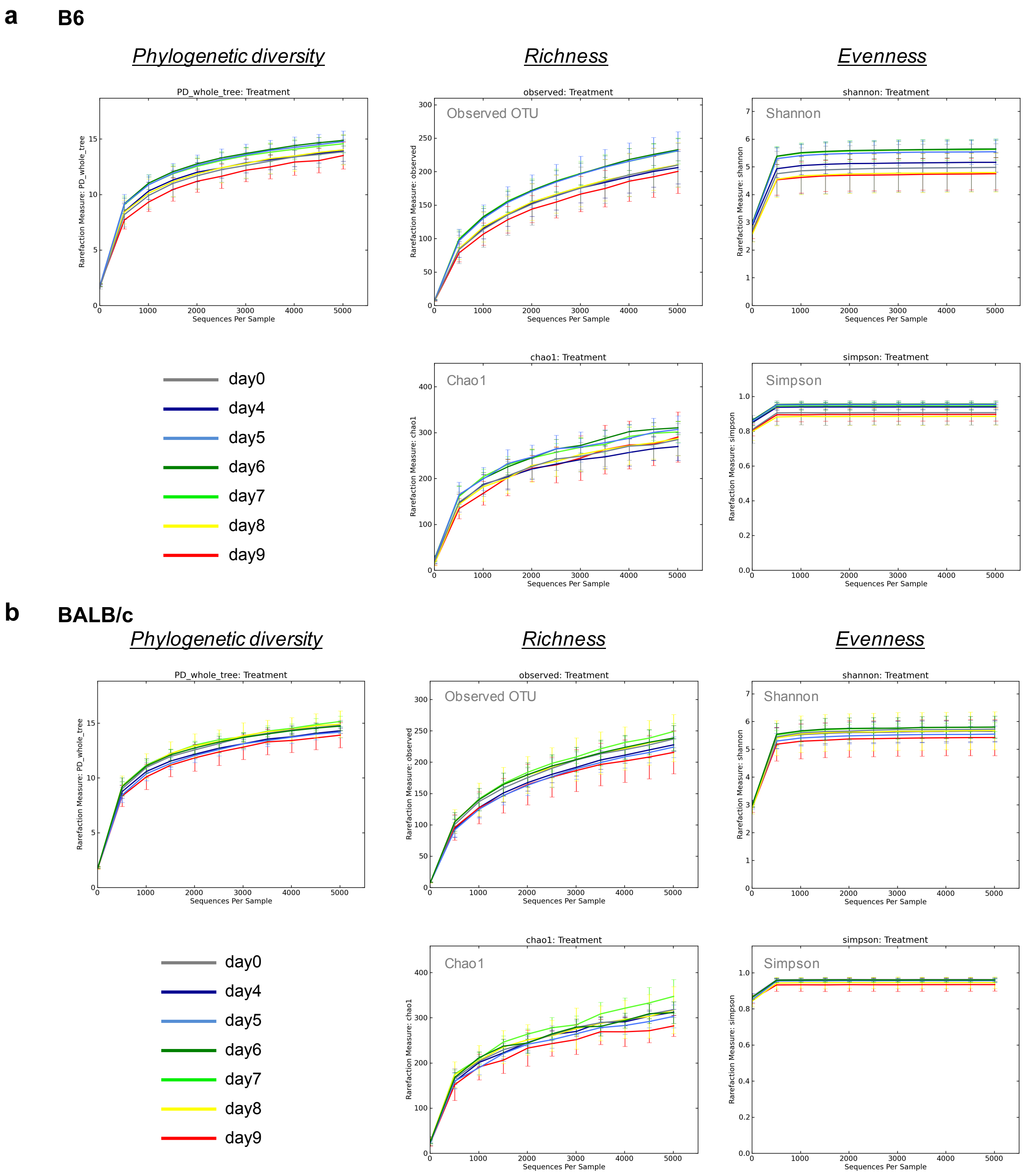

Supplementary Fig. 1 Changes in microbiota diversity following *Pb*A infection. Alpha diversity rarefaction plots of each metric were generated using QIIME pipeline. Values are means ± S.D. from 10 B6 (a) and 5 BALB/c mice (b) the indicated days after infection with *Pb*A.

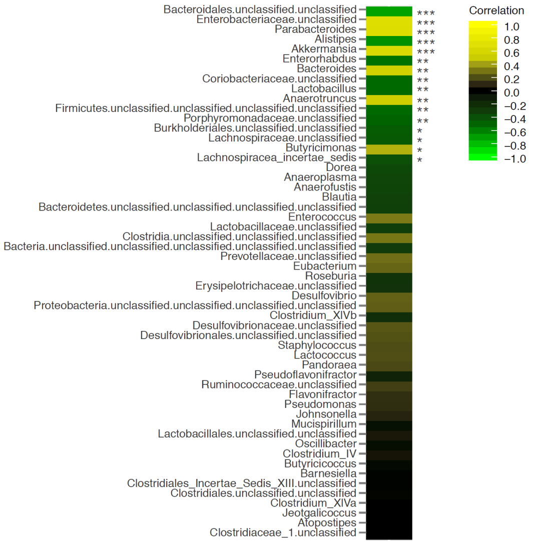

Supplementary Fig. 2. Correlation between genera of bacteria and ECM scores in B6 mice infected with *Pb*A. ECM scores and the abundance of genus levels were co-plotted. The results are shown as for Fig. 6. The genera are listed in order of decreasing strength of correlation. Bright yellow or green indicates a stronger positive or negative correlation, respectively. Statistical analyses were performed using Pearson' s correlation coefficient test. *$p<0.05$, **$p<0.01$, ***$p<0.001$.
